# Influence of the Poly(ethylene Glycol) Methyl Ether Methacrylates on the Selected Physicochemical Properties of Thermally Sensitive Polymeric Particles for Controlled Drug Delivery

**DOI:** 10.3390/polym14214729

**Published:** 2022-11-04

**Authors:** Agnieszka Gola, Maria Kozłowska, Witold Musiał

**Affiliations:** Department of Physical Chemistry and Biophysics, Pharmaceutical Faculty, Wroclaw Medical University, Borowska 211, 50-556 Wroclaw, Poland

**Keywords:** nanoparticles, *N*-isopropylacrylamide, poly(ethylene glycol) methyl ether methacrylate, lower critical solution temperature, anionic initiator, ammonium persulfate, electrical conductivity, controlled drug delivery

## Abstract

Thermosensitive copolymers P1–P5 of *N*-isopropylacrylamide (NIPA) and poly(ethylene glycol) methyl ether methacrylates (PEGMEMs) were synthesized via surfactant-free precipitation polymerization (SFPP) using ammonium persulfate (APS) at 70 °C. The polymerization course was evaluated by the conductivity. The hydrodynamic diameters and the polydispersity indexes (PDI) of P1–P5 in the 18–45 °C range, which were assessed via dynamic light scattering (DLS), were at 18° (nm): 26.07 ± 0.54 (PDI 0.65 ± 0.03), 68.00 ± 1.10 (PDI 0.56 ± 0,02), 45.12 ± 0.57 (PDI 0.51 ± 0.03), 62.78 ± 0.40 (PDI 0.53 ± 0.003), and 92.95 ± 1.56 (PDI 0.60 ± 0.04), respectively. The lower critical solution temperatures ranged from 31 to 33 °C. The electrophoretic mobilities estimated the zeta potential in the 18–45 °C range, and at 18 °C, they were (mV): −4.64 ± 1.30, −6.91 ± 2.67, −5.85 ± 3.17, −2.28 ± 0.30, and −3.60 ± 0.96 for P1–P5, respectively. The polymers were characterized by Attenuated Total Reflectance Fourier-Transform Infrared spectroscopy (ATR-FTIR), H nuclear magnetic resonance (^1^H NMR), thermogravimetric analysis (TGA/DTA), Differential Scanning Calorimetry (DSC), and powder X-ray diffraction analysis (PXRD). Stable amorphous polymers were obtained. We conclude that the length of the co-monomer chain nonlinearly influences the properties of the obtained thermosensitive polymer nanostructures.

## 1. Introduction

Pharmaceutical and biological therapeutic agents are often of limited use due to their short half-lives, poor bioavailability, instability, and them having numerous side effects. Therefore, new drug delivery systems have been developed and investigated to improve the pharmacological performance of the active pharmaceutical ingredients (APIs) [1,2,3].

The methods of drug administration that have been used so far do not fully use the therapeutic properties of the drug substances. The remarkable problem is the insufficient distribution of the drug in the body tissues, resulting in higher initial doses of the drug, what often leads to an increase in the side effects of the therapy. The modern drug form technology should construct drug forms with an API that is released directly to the disease site. It may enable dose reduction, which in turn minimizes the toxic effects on the healthy tissues [4]. The active API carriers may increase the probability of the target site that is reached by the drug and eliminate its excessive distribution, also, in continuous and sustained release systems. The size of the carrier particles is also of high importance. The application of nanoparticles may enhance the rate of effective drug penetration through the biological membranes and increase the stability of it, which results in longer drug presence periods in the circulatory system [5,6,7,8].

Polymers that respond to external stimuli, including specific physiological triggers, are currently being explored as ‘smart’ drug delivery systems [9,10,11]. They are capable of releasing the API in a non-linear response to a stimulus that leads to macroscopic changes in the polymeric structure. The temperature-sensitive polymers, including poly-*N*-isopropylacrylamide (PNIPA), are of great interest due to them having a lower critical solubility temperature (LCST) that is close to the physiological temperature of the human body, which is in the range of 32–34 °C, as well as the ease of controlling and external applying the stimulus in a non-invasive manner [12,13,14]. However, the PNIPA requires that it is modified by the incorporation of additional functional groups in the polymer chain to improve its stability and mechanical properties, i.e., by copolymerization or cross-linking with synthetic or natural substrates [15,16,17,18,19,20]. A significant improvement to the release of the API may be achieved by optimizing the number of hydrophilic and hydrophobic segments of the polymer, and affecting the temperature of the phase transition [21]. Many studies so far have used polyethylene glycol (PEG) substrates for this purpose to improve the biocompatibility, water solubility, and colloidal stability of the polymer, as well as to improve the pharmacokinetic properties of the APIs [22,23,24,25,26,27,28].

Several methods enable us to obtain detailed insight into the physical and chemical properties of the newly developed drug carriers. The electrolytic conductivity of the synthesized systems provides information on the particular stages of polymerization [29,30,31,32]. The proton nuclear magnetic resonance (^1^H NMR) and attenuated total reflectance Fourier-transformed infrared spectroscopy (ATR-FTIR) methods confirm the production of the polymerization product. The thermosensitive polymers in aqueous dispersions require specific evaluations including an assessment of the hydrodynamic diameter (HD), polydispersity (PDI), and zeta potential (ZP) which reveal the possible interactions that occur between the water molecules surrounding the polymer and the hydrophilic groups of the polymers during temperature changes. The thermogravimetric analysis (TGA) and differential scanning calorimetry (DSC) enable an evaluation of the transition temperature, melting temperature, decomposition temperature, and weight loss, which determine the stability and quality of the synthesized product. The powder X-ray diffraction analysis (XRPD) that is applied to amorphous polymeric materials may contribute to the recognition of the material and to the estimation of the degree of crystallinity resulting from impurities and the presence of unreacted substrates or semi-crystallinity.

The aim of the project was to synthesize thermosensitive copolymers based on *N*-isopropylacrylamide (NIPA), and poly(ethylene glycol) methyl ether methacrylate (PEGMEM) co-monomers, that differ in the length of the chain, and to evaluate their physicochemical properties to be applied as potential temperature-triggered drug carriers.

The work continues the former studies of Musial et al. [33] and focuses on physicochemical studies to determine the impact of different chain lengths of the commoner on the characteristic properties of the synthesized thermosensitive polymer structure. The structural properties of the synthesized co-polymers P1–P5 were characterized using ATR-FTIR, ^1^H NMR, PXRD, whereas the thermal properties of the co-polymers were examine by TGA and DSC. The original approach to controlling the polymerization steps was performed via taking measurements of the electrolytic conductivity in a reaction mixture during the entire synthesis process.

## 2. Materials and Methods

### 2.1. Materials

*N*-isopropylacrylamide (NIPA, 99% St. Louis, MO, USA), ammonium persulfate (APS, 98%, Sternheim, Germany), triethylene glycol methyl ether methacrylate (PEGMEM, 93%, average Mn ~200, St. Louis, MO, USA), poly(ethylene glycol) methyl ether methacrylate, (PEGMEM, average Mn ~300, ~500, ~950, ~1500, St. Louis, MO, USA), and dialysis tubing cellulose membrane (MWCO 12,000–14,000 Da St. Louis, MO, USA) were obtained from Sigma Aldrich. The deuterium oxide-NMR solvent was acquired from Merck KGaA, (D_2_O, 99.9 at% D, Darmstadt, Germany). The deionized water (<0.06 μS cm^−1^), was filtered in an HLP 20 system (microfiltration capsule 0.22 μm, Hydrolab, Straszyn, Poland) and met the requirements of the PN-EN ISO 3696:1999 standards for analytical laboratories. All of the chemicals and solvents were used as received without further purification and modification.

### 2.2. Synthesis

The surfactant-free precipitation polymerization (SFPP) method, which was evaluated by Pelton and further developed by other researchers [34], was used to synthesize five thermosensitive co-polymers P1, P2, P3, P4, and P5. The monomer NIPA (~5.0 g) and respective co-monomer PEGMEM (~0.5 g) were polymerized using an anionic initiator ammonium persulfate (APS) in an aqueous environment of 1000 mL volume at 70 °C under a nitrogen atmosphere for 6 h. The deionized water was used as a solvent. The reaction conditions with acronyms of the substrates are listed in Table 1. The molar ratios of the main monomer to the initiator and co-monomers were as: NIPA:APS:PEGMEM (Mn~200)—1:0.05:0.05; NIPA:APS:PEGMEM (Mn~300)—1:0.05:0.04; NIPA:APS:PEGMEM (Mn~500)—1:0.05:0.02; NIPA:APS:PEGMEM (Mn~950)—1:0.05:0.01; NIPA:APS:PEGMEM (Mn~1500)—1:0.05:0.008.

A four-neck, round-bottom 2000-mL flask reaction vessel equipped with an Allihn condenser of 300 mm length including a nitrogen outlet, nitrogen entrance, temperature sensor, conductivity cell of *K* = 1 cm^−1^ and a magnetic stirring bar that was used at 250 rpm was heated in water bath. The required amount of dry sample of the initiator were charged to the reaction vessel that was filled with 900 mL of deionized water and heated to 70 °C and then, continuously stirred and degassed by bubbling nitrogen for ca. 10 min. Next, the monomer and co-monomer were separately dissolved in 50 mL of deionized water, then, mixed and added to the reaction vessel, thus initiating the polymerization reaction.

After six hours of polymerization, the heat was turned off, and the reaction mixture was left for approx. 16 h to cool down to an ambient temperature. Subsequently, 170 mL of each post-reaction mixture was dialyzed for ca. 6 days against 2000 mL of the freshly deionized, stirring water, which was changed once a day, in semipermeable cellulose membrane tubing (MW cutoff 10 kDa–12 kDa, diameter 43 mm). Before each water change, its conductivity was measured. The purification process was finished when the conductivity measurement was ca. 1.3–1.6 µS cm^−1^ in two consecutive water exchange cycles. Immediately after purification, the samples were used for HD and ZP studies, and next, stored at room temperature in dark glass bottles for future use. All of the purified polymer suspensions of ca. 100 mL were placed in sample containers, frozen, and then, freeze-dried by Alpha 1–2 LD (Martin Christ Freeze Dryers, Osterode am Harz, Germany) for 26 h, and stored dry. The dried products of the co-polymers were characterized by the NMR, ATR-FTIR, TG, DSC, and XRPD techniques.

### 2.3. Conductivity Measurements

To determine the conductivity of the reaction mixture during the course of the polymerization reaction at a constant temperature of 70 °C and after synthesis in the cooling process, the conductometer CC-505 (accuracy up to 19.999 mS·cm^−1^ ± 0.1%, from 20.000 mS·cm^−1^ ± 0.25%, Elmetron, Gliwice, Poland) was used. The conductometer was equipped with an EC-60 immersion conductometric sensor with platinum electrodes and glass housing (*K* = 1.0 ± 0.2 cm^−1^, Elmetron, Gliwice, Poland) and a temperature sensor Pt-1000A (0–100 ± 0.35 °C). The conductivity and temperature sensors were constantly immersed in the reaction mixture. The compensation of the temperature was assured manually during the polymerization reaction and automatically during the cooling course one.

### 2.4. Attenuated Total Reflection Fourier-Transformed Infrared Spectroscopy Measurements (ATR-FTIR)

The attenuated total reflectance Fourier-transformed infrared spectroscopy (ATR-FTIR) was performed using a Nicolet iS50 FT-IR spectrometer equipped with an universal ATR sampling accessory that was composed of monolithic diamond crystals (Thermo Fisher Scientific, Madison, WI, USA). The resolution of the wavenumber of radiation recorded using the deuterated L-alanine doped triglycene sulphate detector (DLaTGS) was 4 cm^−1^ ± 0.01 cm^−1^. The transmission ATR-FTIR spectra were obtained in the wavenumber range from 4000 to 400 cm^−1^ by taking the average of 32 scans per sample cycle and after automatically subtracting the background spectra. The reference spectra were recorded using a blank ATR crystal each time after cleaning the ATR module and before applying the sample. The ATR element and pressure clamp were washed several times with methanol and dried. The ATR-FTIR spectra of the substrates in the commercial form and the lyophilized polymerization products were measured at an ambient temperature. A small amount of the solid sample or a drop the liquid sample was placed directly on the flat surface of the monolithic diamond crystal cell. The solid sample was pressed using a clamp with a manual adjustment of the total compression force applied to the sample. The liquid sample was neither covered nor pressed. All of the measurements were performed under the same instrument conditions. The analysis of the ATR-FTIR spectral data was carried out using OMNIC software (Version 9, Thermo Fisher Scientific, Madison, WI, USA).

### 2.5. Nuclear Magnetic Resonance Spectroscopy Measurements (^1^H NMR)

The ^1^H NMR spectra were collected on a Bruker spectrometer with the working frequency of 300 MHz (Bruker, Rheinstetten, Germany). The measurements were carried out at two operating temperatures: 25 °C and 42 °C. The deuterium oxide (D_2_O, δ = 4.68) was used as a solvent for all of the samples. The samples for the NMR measurements were prepared by dissolving about 10 mg of each solid or 0.1 mL of liquid samples in 0.7 mL D_2_O, without subsequent filtration or centrifugation, and placed in NMR tube. The measurements were taken at least 24 h after the sample preparation. The chemical shifts (δ) were expressed in ppm relative to the solvent signal.

### 2.6. Hydrodynamic Diameter (HD) and Polydispersity Index (PDI) Measurements

The hydrodynamic diameter (HD), the distributions, and the polydispersity index (PDI) of the aqueous polymer particles dispersion were measured by dynamic light scattering (DLS), utilizing the Zetasizer Nano ZS ZEN3600 device (Malvern Instruments, Malvern, UK) equipped with the standard red He–Ne laser (4 mW, λ = 633 nm). The light scattering measurements were made by using the sensitive avalanche photodiode detector (APD) which was placed at 173° angle by applying a non-invasive backscattering (NIBS) technology. The light intensity was regulated during the measurement by a laser beam attenuator which was adjusted automatically. The measurements were carried out in an optically translucent polyacrylic disposable DTS-0012 cuvette (Malvern Instruments, Malvern, UK). The cuvette which was filled with 1 mL of the sample, which had been purified by dialysis without any precipitation, and not the diluted polymer dispersion, was placed into the temperature-controlled measurement cell. The sample was equilibrated for 240 s before measurements were taken at every new temperature. The DLS measurements were recorded in steps of 1 °C from 18 to 45 °C. The number of runs in one measurement was adapted automatically in the range of 10–100. The cumulants analysis algorithm was used to estimate the HD and PDI. The methods for calculating the HD and PDI parameters by using applied measurements are defined in the ISO standard documents: ISO 13321:1996E and ISO 22412:2008 [35,36,37]. The values of the refractive index and the viscosity of the water for the dispersant and polystyrene latex which were the materials were adopted as the calculation parameters. The average values that are indicated in the figures of the HD and PDI data were finally obtained from five consecutive measurements which were taken at each temperature. The repeated results were in good agreement. The size distribution was presented by intensity, with a PDI value of 0 for a highly monodispersed standard. The Zetasizer^®^ software (version 7.10) was used to design the custom standard operating protocols (SOPs), which were used on further samples without modifications, and to processed data the from the DLS measurements.

### 2.7. Zeta Potential (ZP) Measurements

The zeta potential (ZP) measurements were performed using a Zetasizer Nano ZS ZEN3600 device (Malvern Instruments, Malvern, UK) based on the laser Doppler electrophoresis technique (laser Doppler velocimetry, LDV) with the Zetasizer^®^ software (version 7.11). The measured electrophoretic mobilities (EM) of the polymer particles in the aqueous dispersion were converted to ZP from the Smoluchowski model approximation to Henry’s equation (f(Ka) = 1.5). The U-shaped plastic (polycarbonate) capillary cuvette—type DTS-1070—with a capacity of 0.75 mL and inbuilt gold-plated copper electrodes (Malvern Instruments, Malvern, UK) was used. The measurements were recorded every one degree in the temperature range of 18–45 °C with an equilibration time of 120 s for each temperature. The average of five measurements at one temperature was taken as the value of the zeta potential.

### 2.8. Thermogravimetric Analysis (TGA)

The thermogravimetric analysis was used to determine the thermal stability of the synthesized nanospheres. The weight loss of the samples as a function of the temperature and time was measured using the TG 209 F1 Libra instrument with an automatic sample changer (ASC) (Erich NETZSCH GmbH and Co. Holding KG, Selb, Germany). The samples were heated from 25 to 800 °C at a heating rate 5.0 °C·min^−1^ under a high-pure nitrogen atmosphere at a flow rate of 50 mL·min^−1^. The thermal decomposition experiment was carried out under non-isothermal heating conditions. The 5.0 ± 0.1 mg of lyophilized material, which is structurally similar to a cotton wool amorphous material, was weighted directly into the standard alumina, Al_2_O_3_, crucibles and carefully compacted with a rammer. The tested material was not grated beforehand. The weight loss of the samples was recorded continuously as a function of the temperature and time with a resolution of 0.1 mg. The TG and DTG curves were recorded and analyzed using the Netzsch Proteus 7.1.0 analysis software (Selb, Germany). The initial (TOnset) and final (TEndset) temperatures of the decomposition process were determined from the intersection of the respective adjacent lines on the TG plots. The temperatures T1, T2, and T3 correspond to the fastest weight loss of the samples at 1st, 2nd, and 3rd stages, respectively, and these were determined from the first derivative.

### 2.9. Differential Scanning Calorimetry (DSC)

The differential scanning calorimeter DSC 214 Polyma (Netzsch, Selb, Germany) equipped with an Intracooler IC70 (Netzsch, Selb, Germany) was used to study the glass transition temperature of the synthesized co-polymers P1–P5. Amounts of the freeze-dried samples of about 3.0 mg were sealed in an DSC aluminum (~25 μL) crucibles with a pinhole in the lid. The empty crucible of the same type was used as the reference. The measurements were performed in a nitrogen atmosphere with a flow rate of 50 mL·min^−1^ and heating/cooling rate of 5.0 °C·min^−1^ over a scanning temperature range from 0 to 230 °C according to a set temperature program of heating/cooling/heating/cooling/heating. The experimental running conditions were: heated to 230 °C, were isothermal 2 min, cooled to 0 °C, and were isothermal 5 min. The analysis of the recorded data was executed using Netzsch Proteus^®^ 7.1.0 analysis software (Netzsch, Selb, Germany). The differential scanning calorimetry was used to determine the glass transition temperatures Tg of the obtained co-polymers P1–P5. The glass transition analysis was based on a determination of the characteristic quantities of the glass transition such as the onset, midpoint, inflection, end set temperature and the glass transition height ΔCp.

### 2.10. Powder X-ray Diffraction Analysis (PXRD)

The X-ray powder diffraction patterns (PXRD) were recorded in the Bragg–Brentano (θ/2θ) horizontal geometry using a Bruker D2 PHASER X-ray diffractometer (Bruker AXS, Karlsruhe, Germany) equipped with a LynxEYE detector. The measurements were performed by applying a Ni-filtered CuKα_1.2_ radiation source (λ = 1.5418 Å). The applied voltage and current were set to 30 kV and 10 mA, respectively. The relative intensity was registered in the 2θ range of 5–70° with a step size of 0.02° and scanning speed 4.0 s/step. The divergence slit was 1.0 mm, and the shutter was 0.5 mm. All of the samples were grinded in a agate mortar, put into the Si low background sample holder, Ø 51.5 mm, with an Ø 20 mm x 0.5 mm sample cavity (Brucker AXS, C79298-A3244-B261, Karlsruhe, Germany), pressed to make a flat surface and measured at 295 K in an ambient atmosphere. The rotation of the sample was 15 min^−1^. The PXRD data were analyzed using the Diffrac.Eva V 3.2 software (Bruker AXS, Karlsruhe, Germany). The average crystallite size of the co-polymers nanoparticles was obtained by an analysis of the peaks widths at the half maximum (FWHD) over a long range of 2 theta, and we applied the Scherrer’s formula used only for medium sizes particles up to about 100 nm [38,39]. D=K · λ/β · cosθ where: *D*—crystallite size; K = Scherrer constant depending on the shape of the crystallite—chosen 0.9 for spherical particles [40]; *λ*—wavelength of X-rays (CuK_α_ = 1,54 Å); *β*—full width at half maximum; *θ*—diffracted angle of the peak.

## 3. Results

### 3.1. Synthesis

**Figure 1:**
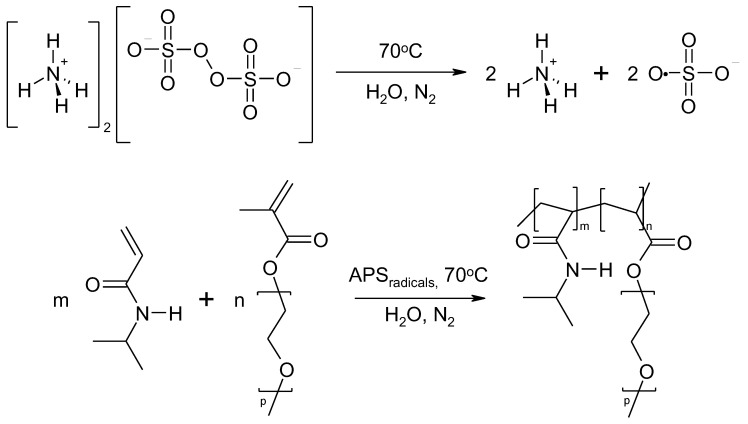
The general scheme of NIPA polymerization with PEGMEM under experimental conditions used in this study.

**Figure 2:**
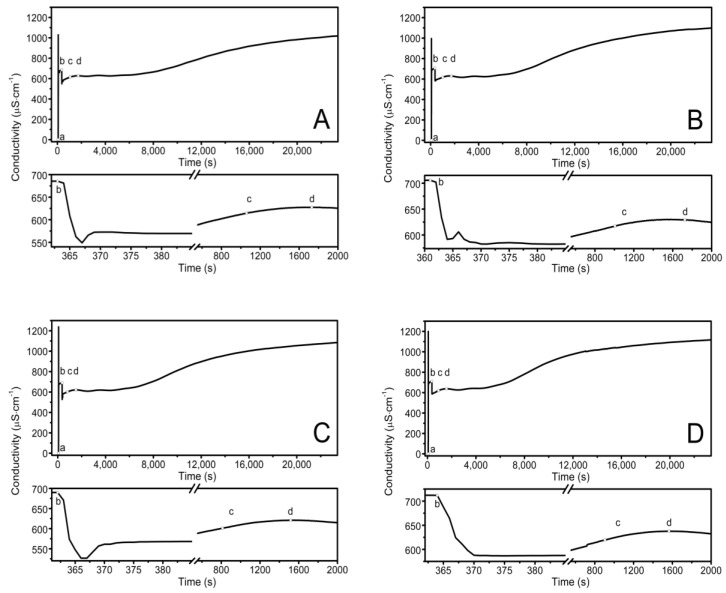

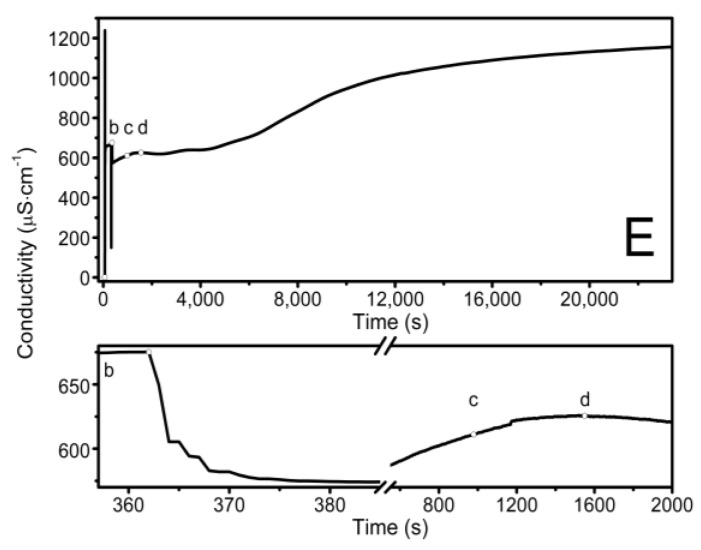
Conductivity in the reaction systems of P1 (**A**), P2 (**B**), P3 (**C**), P4 (**D**), and P5 (**E**) observed over time, over the course of synthesis at T = 70 °C. Point (a) determines the moment of the addition of the initiator—APS, point (b) determines the addition of the aqueous solution of the monomers—NIPA and appropriate PEGMEMs, point (c) determines the beginning of the visible change in the cloudiness of the reaction mixture, point (d) determines the complete turbidity of the reaction mixture.

The observed course of the synthesis of the nano-sized co-polymers P1–P5 is itemized and described in Section 2.2, Synthesis, and Figure 1. The characteristic cloudiness that was observed visually at the macroscopic scale, as the reaction progressed, was revealed to occur at 1059, 1006, 813, 904, and 979 s after the initiating of the polymerization process in the P1, P2, P3, P4, and P5 systems, respectively—Figure 2A–E points (c) and (d).

The synthesized products, the dispersions of P1–P5, were purified via forced equilibrium dialysis (FED) against the deionized water to remove the unreacted substrates and the water-soluble contaminants.

### 3.2. Conductivity Measurements

The conductivity changes over time during reaction for P1–P5 at 70 °C are shown in Figure 2A–E. The polymerization process consists of several consecutive elementary reactions, and the conductivity measurements illustrate the stages of transformations of the substrates into products. The significant differences in the conductivity profiles can be observed at large scaling in a very short period of time lasting 15 s, directly after the introduction of the monomers mixture which initiated the polymerization reaction—the bottom of the diagrams in Figure 2A–E.

**Figure 3:**
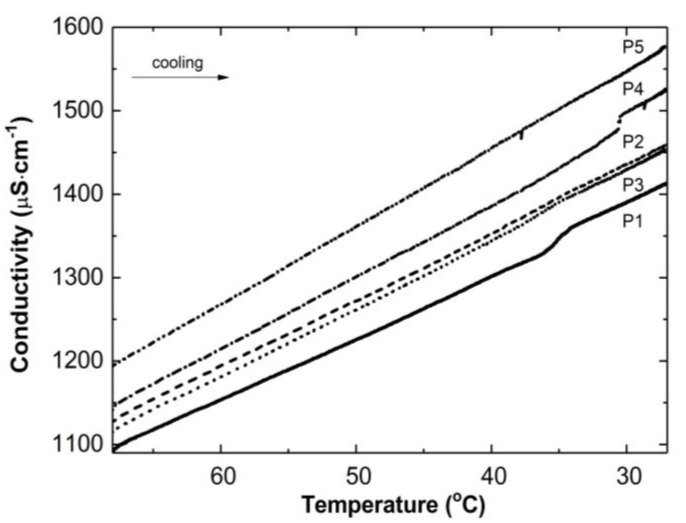
Conductivity changes as a function of temperature in the post-reaction mixtures of P1–P5 during cooling procedure.

Figure 3 illustrates the conductivity profiles of the P1–P5 reaction mixtures as a function of temperature during the cooling of the polymeric systems to room temperature. The conductivity in the studied systems increased almost linearly during the temperature decrease. The visible deflection on the linearity of the conductivity increase was observed in the case of the P1 and P4 systems in a narrow temperature range of 34.2 °C–36.0 °C and 30.5 °C–30.6 °C, respectively. The temperature of the tested reaction mixtures decreased spontaneously and systematically at the ambient environmental temperature.

**Figure 4:**
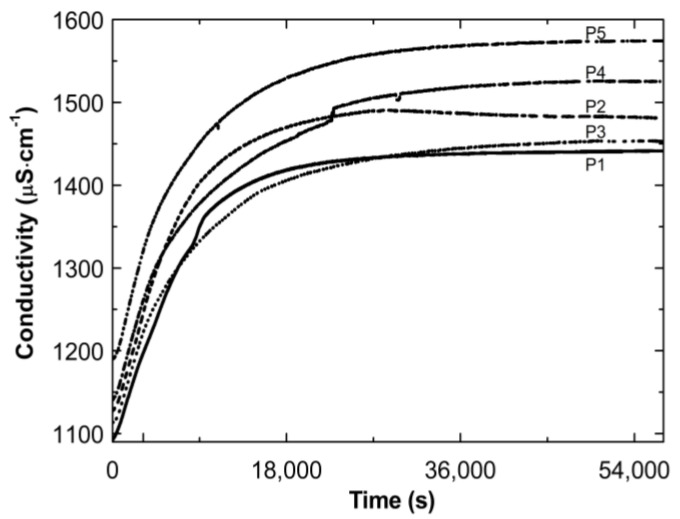
Conductivity changes as a function of time in the post-reaction mixtures of P1–P5 during cooling procedure.

Figure 4 presents the conductivity increase over time during the cooling down of the post-reaction systems P1–P5. The conductivity increased over time. Finally, the conductivity tended to stabilize: by the end of the observation the individual results varied (±4 μS cm^−1^).

### 3.3. Attenuated Total Reflection Fourier-Transform Infrared Spectroscopy Analysis (ATR-FTIR)

**Figure 5:**
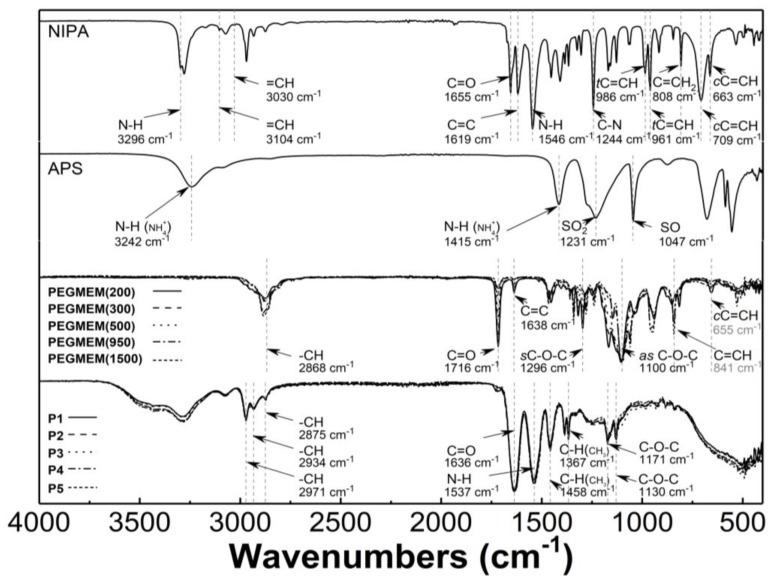
Fourier-transformed infrared spectroscopy with attenuated total reflectance (ATR-FTIR): spectra of monomer—*N*-isopropylacrylamide (NIPA); initiator—ammonium persulfate (APS); co-monomers (PEGMEM); synthesized polymers P1–P5.

Figure 5 presents the typical ATR-FTIR spectra of the monomers—NIPA; five PEGMEMs; the initiator—APS; the synthesized copolymers—P1, P2, P3, P4, and P5. In the spectrum of NIPA, the following bands were observed: at 3296 cm^−1^—valence stretching vibrations of N-H bond [41]; at 3104 and 3030 cm^−1^—low intensity bands corresponding to stretching vibrations of the C-H bond in the vinyl group =CH and =CH_2_ [42,43]; at 2969 cm^−1^—asymmetric C-H stretching vibrations in the C(CH_3_)_2_ isopropyl group [44]; at 1655 cm^−1^—C=O stretching vibrations of the CONH amide I group [45]; at 1619 cm^−1^—stretching vibration of the C=C double bond [46,47]; at 1546 cm^−1^—N-H bending vibration of the CONH amide II group [45,48]; at 1409 and 1368 cm^−1^—C-H deformation vibrations of the isopropyl group [44]; at 1244 cm^−1^—C-N amide III stretching vibrations [49]; at 986 and 961 cm^−1^—trans-C=C-H out of plane deformation vibrations [50]; at 808 cm^−1^—C=CH_2_ out of plane deformation vibrations; at 709 and 663 cm^−1^—cis-C=C-H out of plane deformation vibrations [42,51].

In the spectrum of the APS, all of the expected peaks appeared as follows: at 3242, 1415, 1231, 1047, 679, and 555 cm^−1^, and they were attributed to the N-H stretching vibrations of NH_4_^+^, the N-H deformation vibrations of NH_4_^+^, the SO_2_ stretching vibrations of SO_4_^2−^ group, the S-O stretching vibrations of SO_4_^2−^ group, the SO_2_ bending vibrations of SO_4_^2−^, and the SO_2_ deformations vibrations of SO_4_^2−^ group, respectively [52,53,54,55].

Characteristic bands in the spectra of five PEGMEMs macromonomers were seen at about 2868 cm^−1^—stretching and bending vibration of C-H in methylene group; at 1716 cm^−1^—stretching vibrations of C=O in the carboxylic ester group; at 1638 cm^−1^—stretching vibration of the C=C double bond; at 1296 and 1100 cm^−1^—symmetric and asymmetric stretching vibrations of carboxylic ester C-O-C; at 841 and 655 cm^−1^—out of plane bending vibration of the C=C-H group [56,57,58,59,60].

The ATR-FTIR spectra of the freeze-dried P1–P5 copolymers display peaks that are present at 2971, 2934, and 2875 cm^−1^, which are attributed to the C-H vibrations of isopropyl group; at 1636 cm^−1^—the overlapping bands of the C=O stretching vibrations of the CONH amide I group in NIPA with vibrations of C=O in the carboxylic ester group in the co-monomers; at 1537 cm^−1^—corresponding to the N-H stretching vibrations; at 1458 and 1367 cm^−1^—originating from—C-H deformation vibrations of the isopropyl group; at 1171 cm^−1^ and 1130 cm^−1^, where they are assigned to the C-O-C stretching vibration [61,62].

### 3.4. Nuclear Magnetic Resonance Spectroscopy Analysis (^1^H NMR)

**Figure 6:**
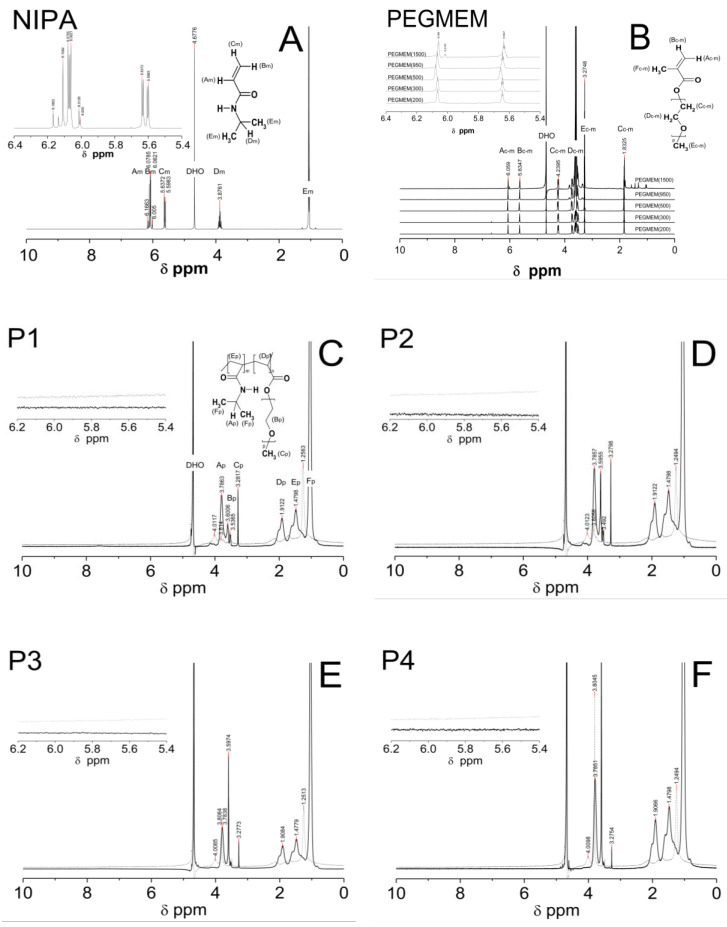

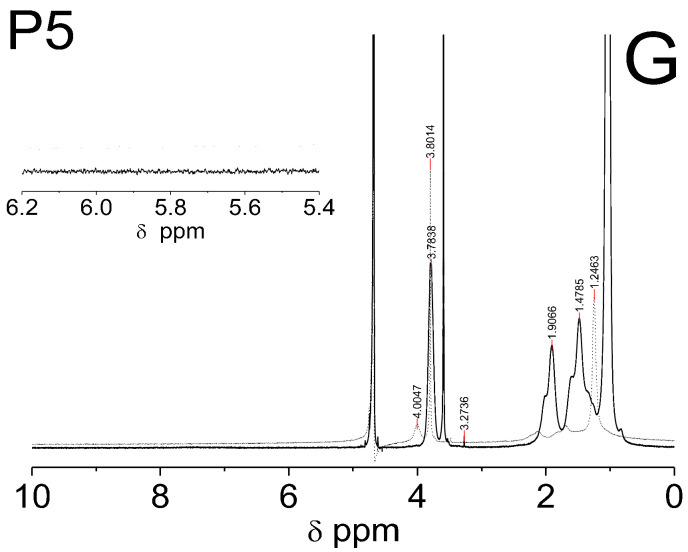
^1^H NMR spectra of monomer NIPA (**A**), co-polymers PEGMEM (**B**), and synthesized polymers P1 (**C**), P2 (**D**), P3 (**E**), P4 (**F**), and P5 (**G**) which were swollen in D_2_O and recorded at 25 °C (solid line) and 42 °C (dashed line). The enlarged areas in the ^1^H NMR spectra present the resonance range of the vinyl protons.

^1^H NMR spectra of the D_2_O solution of the NIPA, five co-macromonomers, and the P1–P5 co-polymers are shown in Figure 6A–E.

All of the characteristic resonances of the different types of protons were assigned to the appropriate chemicals groups and marked equally in the structures of the molecules and in the spectra with successive letters of the alphabet. The signals were listed in order of the largest chemical shifts. In all of the spectra that are presented in Figure 6A–E, there was an observed characteristic peak, which originated from the residual HDO in the NMR solvent D_2_O at a chemical shift of approx. δ = 4.68.

At the ^1^H NMR spectrum of the NIPA, five different signals, indicating the number of groups of equal protons, can be identified. The signals in the ranges of the chemical shifts at δ = 6.18–6.10, δ = 6.09–5.98, δ = 5.67–5.58, δ = 3.95–3.79, and at δ = 1.06–1.04 ppm correspond to the methylene H_Am_ proton of =CH group, the methylene H_Bm_ proton of =CH_2_ group, the methylene H_Cm_ proton of =CH_2_ group, the single proton H_Dm_ of the -CH- group on the *N*-isopropyl, and the methyl proton H_Em_ of the –CH_3_ group on the *N*-isopropyl, respectively [63].

^1^H NMR spectra of the all of the co-monomers PEGMEM contain characteristic following the signals given in ppm of the =CH (H_Ac-m_) at ca. δ = 6.06, =CH (H_Bc-m_), δ = 5.63, -CH_2_OCO (H_Cc-m_), δ = 4.24, -OCH_2_CH_2_- (H_Dc-m_) in the range δ = 3.42–3.87, –OCH_3_ (H_Ec-m_), at δ = 3.27, and –CH_3_ (H_Fc-m_), and at δ = 1.83 [64,65,66].

Figure 6C–E shows the spectra of the co-polymers P1–P5 which were obtained at two temperatures: 25 °C (solid line) and 42 °C (dashed line). The signals that were identified in each of the spectra of the co-polymers P1–P5, which were measured at the same temperature, appeared at identical chemical shifts. The peaks in the spectra which were recorded at a higher temperature are characterized by a lower intensity and are slightly shifted towards higher chemicals shifts (to the left). In Figure 6C–E, the signals at ca. δ = 3.78 ppm (H_Ap_ of the –CH on the *N*-isopropyl), δ = 3.60 ppm (H_Bp_ of the –OCH_2_CH_2_), δ = 3.28 ppm (H_Cp_ of the –CH_3_ of the chain), δ = 1.92 ppm (H_Dp_ of the –CH of the backbone), δ = 1.51 ppm (H_Ep_ of the –CH_2_ of the backbone), and δ = 1.04 ppm (H_Fp_ of the –CH_3_ on the *N*-isopropyl) are observed. In the spectra of the co-polymers P1–P5, which were measured at 42 °C, the intensity of all of the peaks decreases significantly—the peaks at the chemical shifts ca. δ = 4.01 ppm (–CH), δ = 2.12 ppm (–CH of the backbone), and δ = 1.68 ppm (-CH_2_ of the backbone) almost disappear. However, the peaks at the chemical shifts ca. δ = 3.80 ppm (-OCH_2_CH_2_) only in the spectra of P3-P5 and the peak at the chemical shift ca. δ = 1.25 ppm (–CH_3_ on the *N*-isopropyl) in the all of the spectra have a greater intensity than the others do.

In the ^1^H NMR spectra of monomer NIPA and co-polymers P1–P5, no signals in the range of chemical shift at δ = 8.00–7.00 which usually originate from the protons of the NH group on *N*-isopropyl were observed [29,30].

### 3.5. Hydrodynamic Diameter (HD)

**Figure 7:**
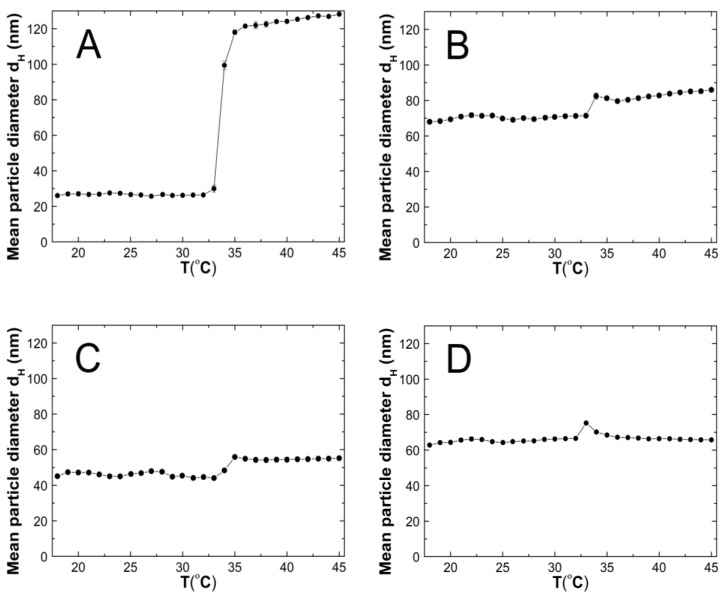

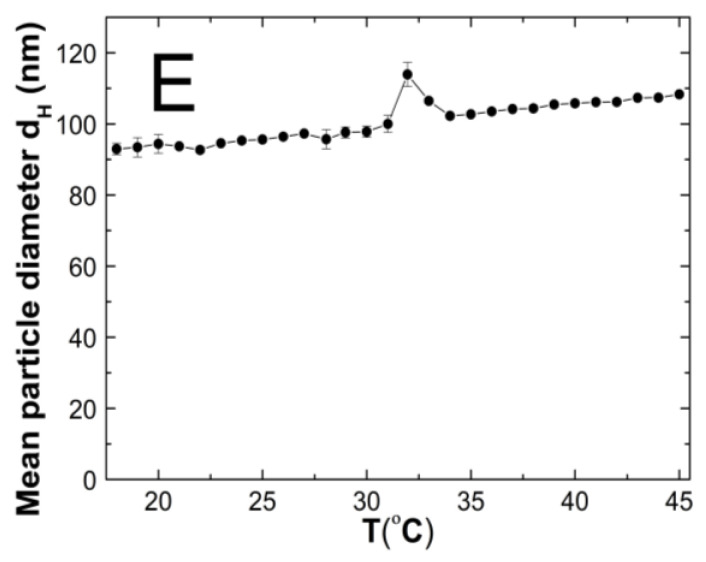
The influence of temperature on the hydrodynamic diameter of the P1 (**A**), P2 (**B**), P3 (**C**), P4 (**D**), and P5 (**E**) samples as determined by dynamic light scattering.

The changes in the hydrodynamic diameters (HD) of the particles in the tested aqueous suspensions of the copolymers P1–P5 as function of the temperature in the temperature range of 18–45 °C are shown in Figure 7A–E. The measurements of the HD were performed via a dynamic light scattering (DLS) technique. The trend of the changes in the hydrodynamic diameter versus the temperature for all of the tested polymers was very similar. The graph profiles clearly indicate three stages of HD levels as defined by the temperature ranges. In the first stage from 18 °C to 32 °C for P1 and P4, at 33 °C for P2 and P3, and at 30 °C for P5, the HD values does not show significant changes and with increasing the temperature, they remained stable with slight deviations. The mean values of the HD in the mentioned temperature ranges were 26.60 ± 0.60, 70.30 ± 0.60, 46.00 ± 0.40, 65.20 ± 0.50, and 95.20 ± 1.27, respectively. The second stage is characterized by a distinct HD growth by 360% in the range of 32–36 °C, by 16% in the range of 33–34 °C, by 26% in the range of 33–35 °C, by 13% in the range of 32–33 °C, and by 14% in the range of 31–32 °C for the polymers P1, P2, P3, P4, and P5, respectively.

The third stage occurred at the temperatures of 36 °C—P1; 34 °C—P2; 33 °C—P3 and P4; 32 °C—P5; these were recognized until the temperature of 45 °C was reached. The estimated lower critical solution temperature (LCST) was 32 °C for P1 and P4, 33 °C for P2 and P3, and 31 °C for P5. All of the measurements of the HD in the entire measuring temperature range were affected by a small standard error, not exceeding 3.5% in most of the cases and 6.2% in one of the HD values.

**Figure 8:**
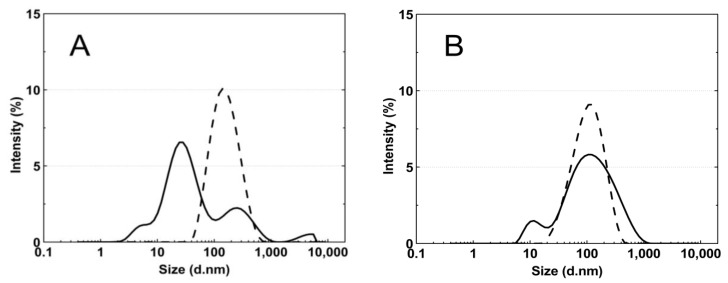

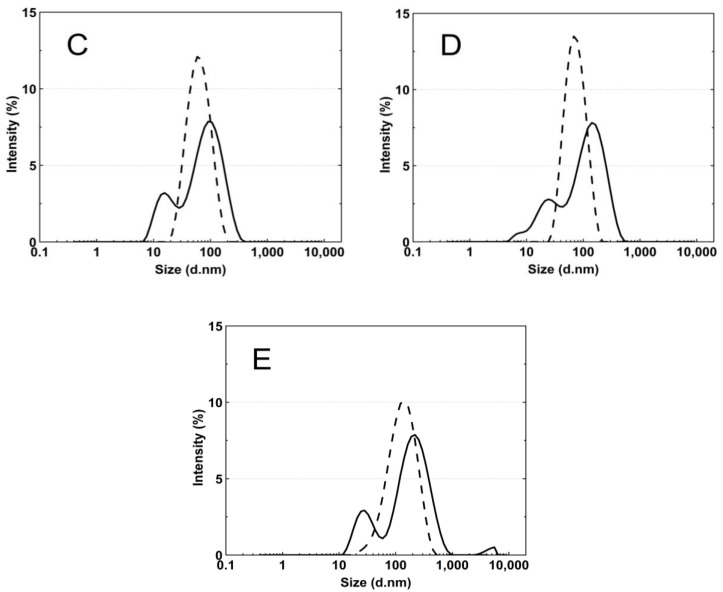
The particle size distributions by intensity for P1 (**A**), P2 (**B**), P3 (**C**), P4 (**D**), and P5 (**E**) dispersions at 18 °C—solid line; 45 °C—dash line; these were obtained by dynamic light scattering (DLS) analysis.

Figure 8A–E presents the standard plots of the size distributions of the P1–P5 nanoparticles at two temperatures, 18 °C and 45 °C. The size distribution corresponds to the most intense peak.

### 3.6. Polydispersity Index (PDI)

**Figure 9:**
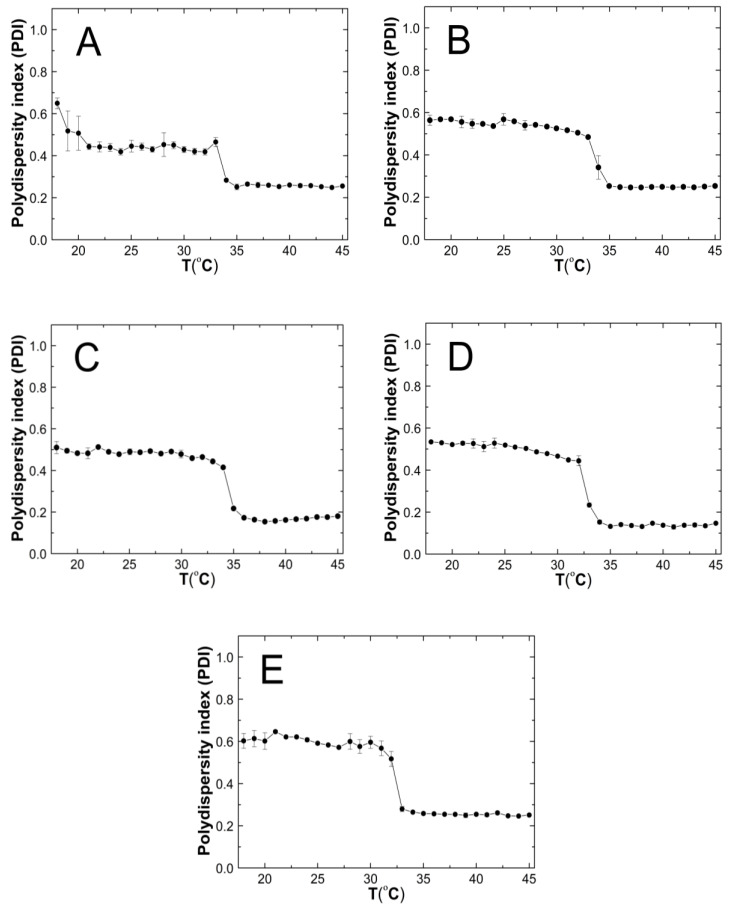
The influence of temperature on the polydispersity index (PDI) of P1 (**A**), P2 (**B**), P3 (**C**), P4 (**D**), and P5 (**E**) samples as determined by dynamic light scattering.

Figure 9A–E shows the results of the polydispersity index (PDI) measurements for the purified P1–P5 co-polymers aqueous suspensions as a function of temperature in the range of 18 °C–45 °C. The PDI values remains approximately stable, with it not exceeding 0.65. Over the temperatures that have been mentioned, significant PDI decreases were noticed. All of the measurement points in the entire temperature range were characterized by a very small measurement error.

### 3.7. Zeta Potential (ZP)

**Figure 10:**
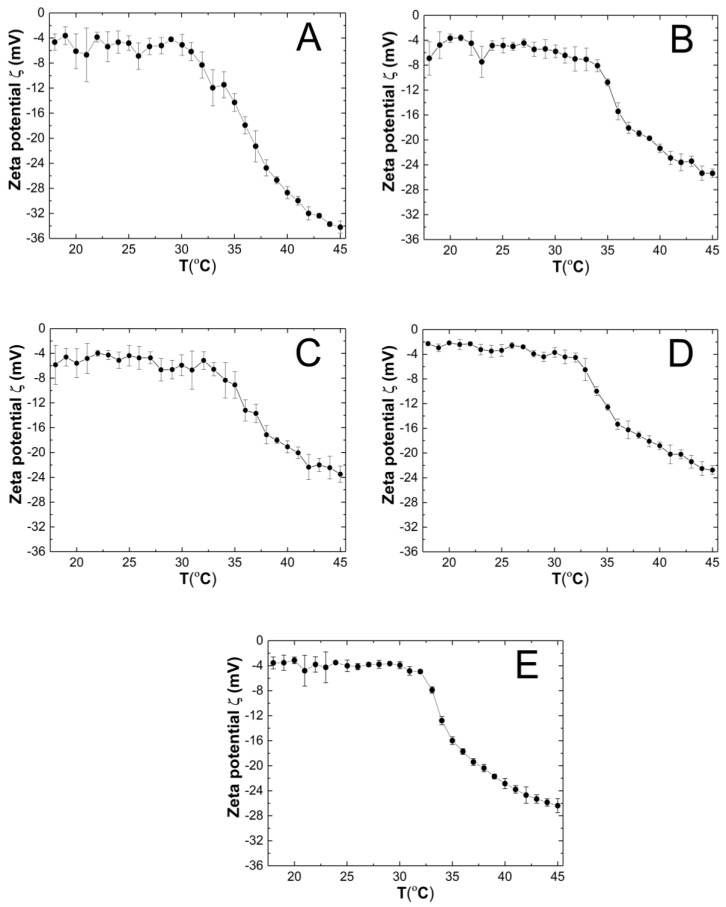
The influence of temperature on zeta potential (ZP) of the P1 (**A**), P2 (**B**), P3 (**C**), P4 (**D**), and P5 (**E**) samples as determined by electrophoretic mobility.

The course of the changes in the zeta potential (ZP) of the P1–P5 purified co-polymer dispersions as a function of temperature in the range of 18–45 °C is shown in Figure 10A–E. The measured samples were not buffered, and their pH which were measured at ca. 22.5 °C were 4.4, 4.8, 4.9, 4.8, and 4.7 for the P1, P2, P3, P4, and P5 polymer dispersions, respectively. Throughout the entire measurement temperature range of 18–45 °C, the ZP values were negative. In the all of the variants of the tested co-polymer dispersions, an increase in the temperature induced the evidence linear decrease in the ZP. At 45 °C, the ZP the absolute values were measured as 34.20 ± 0.97, 25.36 ± 0.68, 23.48 ± 1.31, 22.74 ± 0.70, and 26.38 ± 1.13 mV for P1, P2, P3, P4, and P5, respectively.

### 3.8. Thermogravimetric Analysis (TGA)

**Figure 11:**
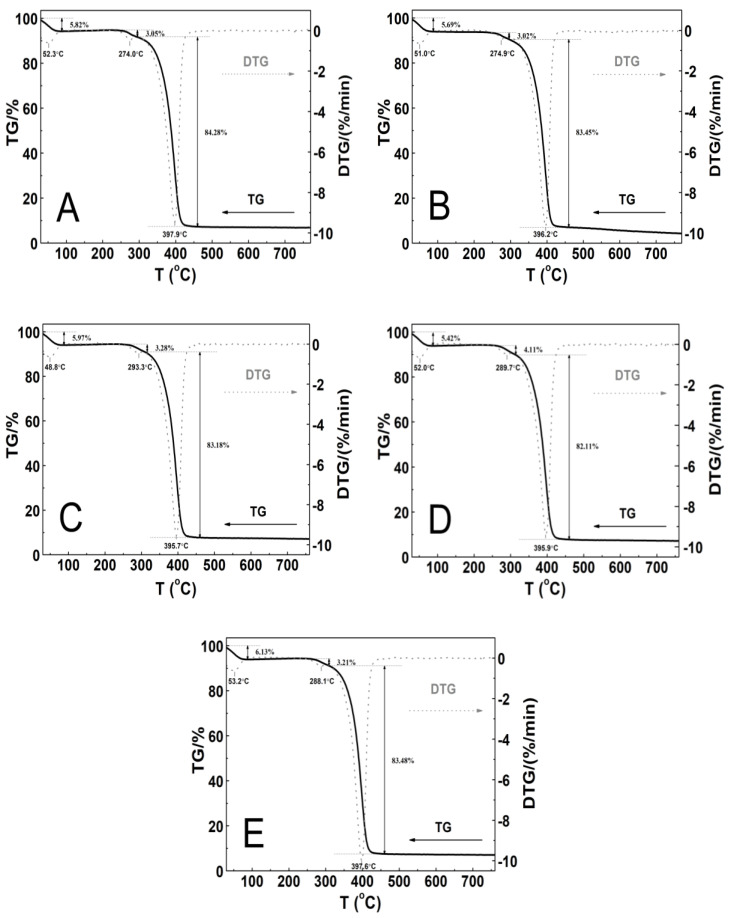
The thermoanalytical curves TG—solid line; DTG—dashed line; these were obtained at the heating rate β = 5 °C min^−1^ in a nitrogen atmosphere at 50 mL min^−1^ for polymers P1 (**A**), P2 (**B**), P3 (**C**), P4 (**D**), and P5 (**E**).

The thermal stability of the tested polymeric material was investigated by measurements of the mass changes in the temperature range from 25 to 800 °C. The thermogravimetric analysis (TGA) and first derivative thermal analysis (DTA) plots are gathered in Figure 11A–E.

The thermographic plots demonstrate similar three-stage mass loss profiles and thermal decomposition patterns for all of the P1–P5 systems. At the initial stages of the thermal degradation from 30 to 90 °C, the mass loss varied within the range from 5.4 to 6.1%. Between 240 and 310 °C, a very small mass loss from 3.0 to 4.1% was observed. The greatest mass loss, ca. 83%, was occurred in the temperature range of 295–460 °C. Above 460 °C, up to the final temperature, the weight loss was slow and negligible. By the end of the process, the residual mass was approximately 4.4% for P2 and 7.1% for P1, P3, P4, and P5 of the initial mass. The recorded DTG curves were characterized by three peaks with the minimum ones being in the temperature ranges of 48.0–53.5 °C and 274.0–293.5 °C and between 395.0 and 398.0 °C. The thermal decomposition of all of the polymers at 760 °C was complete over 90% as the residue amount ranged from 4.0 to 7.2%. Table 2 summarizes the results of the TG and DTG curve analyses for all of the five tested nanopolymers.

### 3.9. Differential Scanning Calorimetry Analysis (DSC)

**Figure 12:**
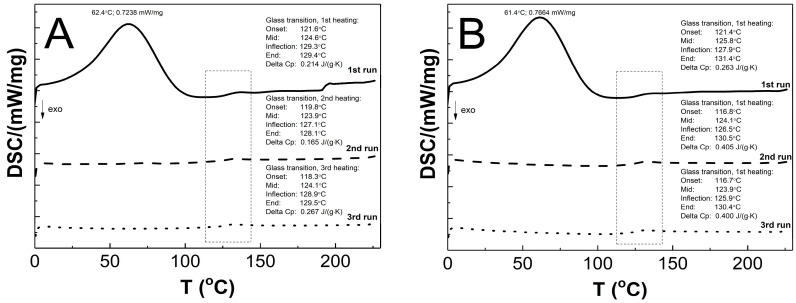

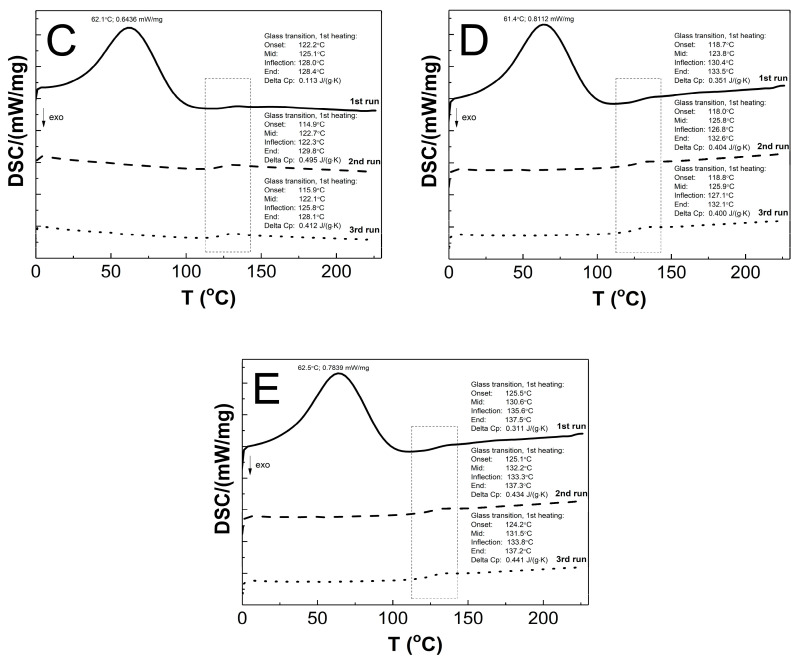
The DSC heating thermograms of 1st (solid line), 2nd (dashed line), and 3rd (dotted line) heating runs for polymers P1 (**A**), P2 (**B**), P3 (**C**), P4 (**D**), and P5 (**E**) at the heating rate β = 5 °C min^−1^ in a nitrogen atmosphere at 50 mL min^−1^.

The DSC thermograms in Figure 12A–E present the thermal plots of the runs of heating: first—solid line; second—dashed line; third—dotted line. In the all of the measurement curves of the 1st run of heating, the broad peak reflects an endothermic effect, and also a small endothermic step was detected. On the measurement curves from 2nd and 3rd runs of heating, no endothermic peak was visible, and just a single step in the endothermal direction was detected. The T_g_ values that were measured for the P1, P2, P3, P4, and P5 co-polymers of 2nd and 3rd runs were similar. The glass transition temperatures of 1st/2nd/3rd heating runs were 124.6/123.9/124.1, 125.8/124.1/123.9, 125.1/122.7/122.1, 123.8/125.8/125.9, and 130.6/132.2/131.5 °C for P1, P2, P3, P4, and P5, respectively. The studied samples P1, P2, P3, P4, and P5 had, accordingly, a ∆Cp of 0.214/0.165/0.267, 0.263/0.405/0.400, 0.113/0.495/0.412, 0.351/0.404/0.400, and 0.311/0.434/0.441 J·g−1·K−1 in the 1st/2nd/3rd heating runs.

### 3.10. Powder X-ray Diffraction Analysis (PXRD)

**Figure 13:**
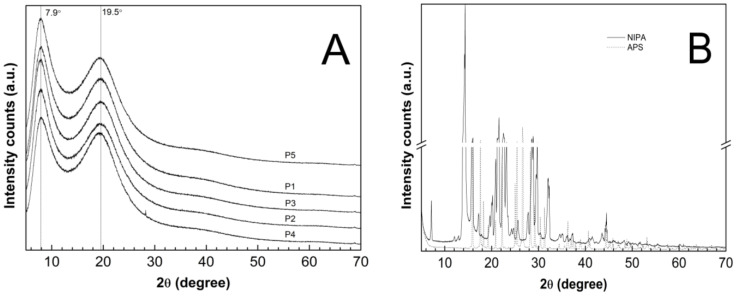
Powder X-Ray diffraction patterns of (**A**) synthesized co-polymers P1–P5; (**B**) monomer—NIPA; initiator—APS.

The PXRD patterns were recorded on the lyophilized and powdered samples of the synthesized P1–P5 co-polymers—Figure 13A—and on the pure and commercial samples of NIPA and APS—Figure 13B. The diffraction patterns of the monomer (solid line) and the initiator (dotted line) had characteristic sharp and intense crystalline peaks between 5° and 70° 2θ. The dominant peaks for the NIPA sample centered at 2θ values of ca. 14.33°, 21.56°, and 22.55°. At the PXRD patterns of the pure APS, three major peaks can be perceived at 26.60°, 15,89°, and 17.61° 2θ of much higher intensity than the others. The XRD profiles of the P1–P5 co-polymers exhibit two pronounced peaks with low intensity diffraction at 2θ values of approximately 7.90° and 19.50°.

The evaluated average crystallite sizes were 1.85 nm for P1, 1.87 nm for P2, P4, P5, and 1.92 nm for P3. The parameters like the peak position, FWHM, and intensity which were obtained by the analysis of the diffraction lines of the P1–P5 co-polymers are listed in Table 3. All of the parameters were determined after subtracting the baseline.

## 4. Discussion

### 4.1. Synthesis

The observed course of the synthesis in the main stages of the process was consistent with the experiments that have been reported by other researchers [67,68,69,70,71], however, detailed differences were identified when we were using conductivity measurements. A thermoshrinking type of transition was observed during each synthesis, in which the collapse of the nanoparticles of the created polymer occurred in response to the influence of the temperature when it was higher than the LCST, which is manifested by the turbidity of the reaction mixture. No linear dependence of the rate of the onset of the appearance of turbidity versus the chain length of the PEGMEMs was observed. The turbidity retreated after the cooling of the reaction systems to the room temperature. The gravimetric data of the mass of the product which were obtained from the synthesis reflect the mass product which was included in 100 mL of dialyzed reaction mixture. One hundred mL of the resulting solutions were freeze-dried to obtain 0.42769, 0.43140, 0.48625, 0.42961, and 0.50008 g of solid, white-colored, cotton wool-like consistency P1, P2, P3, P4, and P5 co-polymers, respectively.

### 4.2. Conductivity

The polymerization process consists of several consecutive elementary reactions, illustrating the stages of transformation of the substrates into products. In line with our previous research [29,30,31,32], continuous measurements of the conductivity of the reaction mixture during polymerization can provide the necessary data to determine the onset and duration of the individual polymerization steps to be determined.

The introduction of the APS initiator into the solvent environment, which at the temperature of 60–70 °C easily decomposed into anionic radicals, resulted in a rapid increase in the conductivity of the tested systems—Figure 2A–E, point (a). The first very high conductivity reading may reflect the rapid formation of initiator decay products (k = 10^−4^–10^−6^s^−1^) [72] or can be related to the local concentration of the substance as the initiator was introduced in a crystalline form. Then, the stabilization of the conductivity at a constant level with a slight uptrend could indicate that some of the radicals which were formed underwent recombination, disproportionation, or solvent-mediated reactions. The diffusion processes in liquids tend to keep adjacent molecules close together for finite periods of time. As the “meeting” time lengthens, the surrounding solvent molecules can take part either in further re-collisions or in the entrapment of the reacting molecules in the so-called cage. The cage effect can be considered to be unfavorable because before escaping from the cage, the radicals can react with each other, self-terminate, or react with the cage-forming particles to form irreversibly inactive products, thus leading to a reduction in the concentration and effectiveness of the radicals that are involved in the polymerization initiation process [73,74,75,76,77]. The preservation of the products of the initiator, which decomposed for 10 min only when they were in contact with the solvent, as was the case in the present studies, favors the formation of the cage effect, but it is a necessary procedure for the removal of oxygen molecules, which is a side product of the radical formation reaction in an aqueous environment at high temperatures. However, the amount of initiator that was used and the preservation time for the introduction of the monomer mixture were the same in all of the performed reactions P1–P5. Therefore, it may be assumed that each reaction had the same starting conditions, and possibly, an occurring cage effect should not influence the comparison of the individual syntheses.

The second distinctive change—the decrease in conductivity—occurred after the addition of the mixture of aqueous monomers solutions to the reaction system (Figure 2A–E, point (b)). The polymerization process began, and it was associated with the attachment of the active centers resulting from the decomposition of the initiator to the molecules of monomers with unsaturated bonds. The addition reactions ran almost immediately, hence, there was a short process time. Additionally, in the copolymerization reaction, several addition reactions usually take place in parallel because two monomers compete with each other for the attachment to the active sites of the initiator radicals.

The other visible change in the close-up may be related to the escape of the radicals from the cage due to the addition of monomers to the system, and above all, to the diffusion of two macroradicals towards each other from two random positions and the rearrangement, rotation, and configuration of the reaction sites of the molecules in a manner that is appropriate to the course of the reaction. The particles of smaller size and less dynamic stiffness moved faster, resulting in the visible short-term increase in conductivity in the P1–P3 systems (Figure 2A–C, bottom graphs at ca. 365 s). The further established stable conductivity level may be related to the equilibration of the rates of the formation and disappearance of the active centers. This may indicate the early stage of the polymerization process in which the active centers are formed, and propagation is not yet advanced. The rates of the initiation, propagation, and termination processes regulate the concentration of the active centers during polymerization, which may be constant, increasing, or decreasing.

Thereafter, a number of possible elementary reactions in the polymerization process can take place, increasing with the increase of the involved monomer particles. The reactivity of the active centers of individual monomers determines the addition of the monomer to its own or extraneous radical. The chain propagation occurred, which was reflected by another slow increase in conductivity (Figure 2A–E at ca. 4000 s). Due to the amphiphilic nature of the monomers, the forming oligoradicals orient themselves into micelles, inside which the monomer particles can be captured, and so-called polymer–monomer particles are formed. The size of the micelles changes until it reaches a thermodynamically stable state. As the reaction proceeds, the trapped monomer molecules or other species may diffuse out of the micelles, thus increasing the conductivity of the system. The temperature at which the reaction is carried out, when exceeding the phase transition temperature, promotes the shrinkage of the polymer spheres and precipitation in the aqueous phase. The shrunken products may be ejected from the precipitate or remain partially unreacted. The inactive monomer molecules or oligopolymers with small number of units may be stored in the precipitate. The storage phenomenon was confirmed by the conductivity measurements of the reaction mixtures during the cooling procedure (Figure 3). In the process of the lowering of the temperature of the post-reaction mixture, the collapsed polymer particles returned to their developed form, which facilitates the diffusion of the trapped compounds outside of the polymeric nanosphere, resulting in a gradual increase in conductivity. The conspicuous deviations from the linearity in the profile of the changes in conductivity vs. temperature for P1 at the temperature range of 34.2 °C–36.0 °C characterize the moment of the phase transition (Figure 3). These results correspond well with the DLS measurements for P1 dispersion, whereas in the case of the P4 post-reaction system, the recorded deviations from linearity can be regarded as artifacts resulting, for example, from a voltage drop (Figure 3). The increasing exponential function describes the changes in conductivity in the studied P1–P5 post-reaction systems (Figure 4).

### 4.3. ATR-FTIR

The main confirmation of the copolymerization of NIPA and PEGMEMs is the absence of the carbon–carbon double bond absorption peaks in the spectra of the P1–P5 polymers—Figure 5. Due to the overlapping bands in the region of the strongest stretching vibrations of the unsaturated C=C band for NIPA and PEGMEM in the spectra of the polymers, the additional confirmation of the copolymerization is the appearance of characteristic peaks in the spectra of the polymers P1–P5, originating from the most important functional groups of monomers. Therefore, in the polymers spectra, the bands at 1636, 1537, 1171, and 1130 cm^−1^ due to the vibration of the C=O, N-H, and C-O-C groups are visible. Moreover, the increase in the intensity of the peaks in the region at 2970–2875 cm^−1^ and at 1636 and 1537 cm^−1^ indicates an enrichment the copolymer structure with the C-H, C=O, and N-H groups. This results also suggest that the synthesis of poly (NIPA-co-PEGMEM) was successfully carried out.

### 4.4. ^1^H NMR

The polymerization reaction was confirmed by a ^1^H NMR analysis primarily via comparing the resonance range of the protons of the vinyl group in the spectra of the substrates and polymerization products. The complete disappearance of the signals in the spectra of the co-polymers P1–P5 (Figure 6C–G), which were derived from protons of the vinyl group NIPA and PEGMEM molecules has been broken, and the polymerization reaction occurred successfully under the experimental conditions, and in the tested samples, there were no unreacted substrates. The presence of characteristic peaks, originating from the protons of the oxyethylene group (δ = 3.60 ppm), in the spectra of the co-polymers P1–P5, may certify the incorporation of PEGMEM in the polymer network and confirm the synthesis of the co-polymers NIPA-co-PEGMEM (Figure 6C–G).

The shifts of all of the proton signals, except the signal from DOH, in the spectra of the co-polymers P1–P5 recorded at the temperature above the LCST of PNIPA, which is ca. 32 °C, may be caused by the breaking of the hydrogen bonds at higher temperatures and the changing of the interactions between the water molecules and the hydrophilic groups of the polymer chain [78,79]. However, the reduction of the intensity of these peaks can be attributed to the hindrance of the internal rotation and loss of mobility of the polymer chain, which at higher temperatures collapses and transforms into the structure of the globule, resulting from the collapse and transformation of the polymer network into a structure of the globule at higher temperatures [80,81]. Furthermore, based on the analysis of the peaks intensity, it can be concluded that at 42 °C, the studied co-polymers chains spontaneously collapsed and changed their conformations so that the chemical groups –CH_3_ on the *N*-isopropyl were oriented upward to the surface of the coiled form. In the case of P3–P5, also, a chemical group -OCH_2_CH_2_ appeared to orientate upward to the surface (Figure 6E–G, dashed line), whereas the rest of the chemical groups in the P1–P5 polymer backbones were located inside the globule (Figure 6C–G, dashed line).

The lack of signals from the proton of the NH group on *N*-isopropyl both in the spectra of the monomer NIPA and the P1–P5 co-polymers is due to the easy exchange of protons from the –NH group with deuterium in D_2_O. This phenomenon also suggests that there was no rigid configuration of the obtained polymeric network and that a relatively easy exchange of protons occurred throughout the entire structure [82].

### 4.5. HD

**Figure 14:**
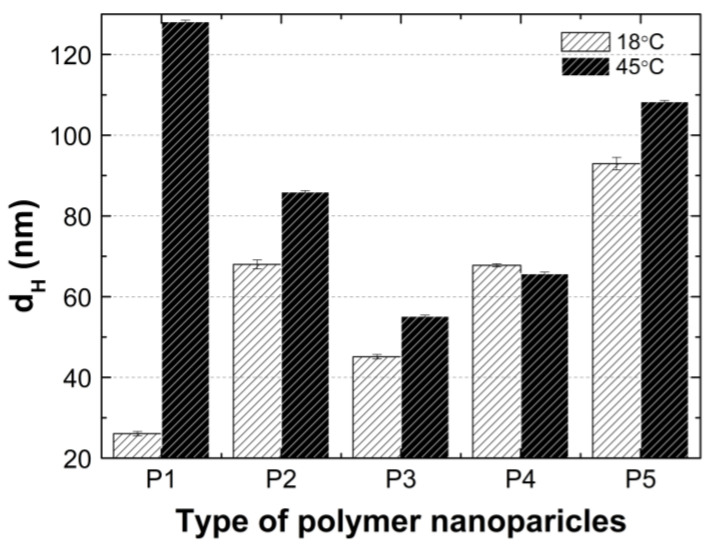
Hydrodynamic diameters of P1–P5 measured at 18 °C and 45 °C.

According to the graph in Figure 14, at 18 °C, the size of the hydrodynamic diameter increased linearly with the growth of the PEGMEM chain in order for polymers P1, P3, P4, and P5. The particle size of the polymer P2 deviates upward from a linear relationship as shown. The value of the HD of the P2 polymer particles is more than 50 percent greater than the HD value of the P3 polymer particles, moreover, is they are comparable to the HD of P4. The predictable HD value of the polymer particles P2 should be higher than that of the polymer P1 and lower than the HD of the polymer P3. Possibly, the chain length of PEGMEM 300 and the formed layer on the nanoparticle do not sufficiently prevent the attraction between the nanoparticles [83]. Moreover, the disruption of the proportion of the hydrophobic to the hydrophilic elements of the molecule may lead to a higher degree of aggregation at 18 °C [84].

The HD values of the tested polymer particles, except P4, are greater at 45 °C than they are at 18 °C (Figure 14). The increase in the temperature causes the dehydration of the oxyethylene chains and may reduce the Coulomb repulsion between the hydrophilic groups, thus leading to the formation of large aggregates [85]. The size of the aggregates with the increase of the PEGMEM chain length decreased for polymers P1–P3 and increased for polymers P4 and P5. The reduction in the size of the copolymer aggregates in the P1–P3 pattern may be due to the increasing number of relatively short PEGMEM chains in these macromolecules and the resulting loose packing of the nanospheres. The HD value of the P4 polymer decreased, and the P5 polymer slightly increased when it was compared to the HD that was observed at 18 °C. These suggests that the structures with a lower number of aggregations were obtained with better protection of the internal hydrophobic groups by the long hydrophilic chains of PEGMEM, which prevented their aggregation into structures with a higher aggregation number [86,87].

According to the particle size distribution analysis of the dispersion of the P1–P5 co-polymers at 18 °C, at least two poorly separated populations were present (Figure 8), however, at 45 °C, a monomodal and quite narrow size distribution was observed. The increase in the temperature above the LCST in this case facilitated the aggregation and the formed colloidally stable aggregates—mesoglobules [88,89]. This is related to the loss of entropy by the system, which leads to a specific energetic equilibrium of the system increasing at higher temperatures [90]. The evolution of the size distribution from multimodal at 18 °C to monomodal at 45 °C may indicate the aggregation and further coagulation of the nanoparticles [91].

### 4.6. PDI

Above the phase transition temperature, the PDI values did not exceed 0.26—Figure 9A–E. This indicates that an increased temperature favored the formation of systems with lower polydispersity and higher size uniformity. The co-polymers P1, P2, and P5 possessed PDI values that were similar to each other, of ca. 0.26, whereas P3 and P4 achieved the lowest PDI values of approximately 0.17 and 0.14, respectively. These discrepancies may be attributed to the different distributions as well as the number of hydrophobic groups—constituting the micellar core and hydrophilic groups—thus, forming the crown and the interaction between themselves and the solvent at a higher temperature, which affects the formation of more or less homogeneous systems [92]. The polymers with the greatest variation between the initial and final values of the phase transition temperature (∆T) have the lowest PDI values at higher temperatures. This may indicate that the P3 and P4 co-polymers below the LCST in the aqueous solutions exist in the form of small aggregates wherein, their decay rate exceeds the growth rate, and the main aggregation process could only take place above the LCST. Polymers P1, P2, and P5, when they were below the LCST tended to form larger, more stable aggregates, and when they were above the LCST, their aggregation was more intense.

### 4.7. ZP

The ZP values for samples P1–P5 throughout the entire temperature range of 18–45 °C reached negative values (Figure 10A–E), presumably due to the presence of sulfate groups from the APS initiator, thus terminating the polymer chains. The system stability with the PZ range from 2.20 to −34.20 mV is considered to be low [93]. Only the P1 polymer dispersion at 45 °C would be a stable system with a PZ of −34.20 mV. However, the relatively low standard errors of the measurements may suggest a kind of equilibrium at each temperature for the P1–P5 systems. In this case, the steric effect on the stabilization of dispersion has a greater influence when we are comparing it to the electrostatic interactions. In some cases, the presence of a weak attracting interaction with a mild effect of repulsive forces which register as a low ZP may be sufficient for the stability of the system [94]. Moreover, the presence of nonionic PEGMEM chains in the molecule should increase the stability of the system through the formation of the coatings, but also reduce the ZP values [95,96]. On the basis of the obtained results, no significant and expected correlation between the PEGMEM chain lengths and the ZP value of the synthesized nanoparticles was observed. However, in the case of P1—Figure 10 (A)—it is likely that the short PEGMEM chain may provide an insufficient particle coverage, and the shielding of negative groups may not be as effective, especially at higher temperatures, where the thermo-sensitive particles shrink and transfer the large, negatively charged sulfate groups to the surface. The phase transition temperature may indicate the beginning of the formation of large aggregates, which is favored by the increase in the kinetic energy of the system by the temperature [97].

### 4.8. TG

The P1 and P2 polymers (Figure 11A–B) exhibited thermal stability up to 240 °C, while P3, P4, and P5 (Figure 11C–E) exhibited thermal stability up to 250 °C. In the first stage, the almost 6% decrease in the weight of the tested samples may have occurred via the evaporation of adsorbed moisture and trapped water which was not removed by the freeze-drying process, the release of bound water, or first order phase transitions, e.g., melting [98,99,100]. The second stage represented of nearly 4% of the mass loss, and this may be attributed to the degradation of the ammonium sulfate group at the polymer chain end, originating from the initiator, into ammonia and sulfuric acid [101,102], as well as the thermal degradation of the hydrophobic side chains [102]. The major mass loss of about 80% can be assigned to the decomposition of the amide group and the degradation of the macromolecular chain of the copolymer [103,104].

The DTG profiles for the synthesized copolymers P1–P5 closely correspond to the recorded typical TG curves. The comparison of the TG and DTG data between the P1–P5 copolymers revealed that there were not very large differences between the characteristic decomposition temperatures as indicated by respective DTG peaks, and as well as this, it points to there being an almost identical decomposition behavior and similar reactivity between the tested samples. The influence of the varied length of the polyoxyethylene glycol chain on the thermal behavior of the synthesized polymers P1–P5, contrary to the expectations, was not so considerable and additionally, it was nonlinear. There was small shift of the temperature of the maximum rate of the decomposition for the polymers P1–P3 towards lower values, and for the P4–P5 polymers, this was towards higher values.

### 4.9. DSC

As shown in Figure 12A–E, the endothermic deviations from the baseline representing heat flux, which are displayed as peaks and were detected at ca. 62 °C on the DSC curves during the 1st heating stage, are most probably related to the heat flow which was used to remove the water that was retained in the polymers network and the residual moisture during the DSC experiment, which is reflected in the TG research. The DSC studies show that all of the tested polymers exhibit glass transition at one temperature interval which may indicate structural regularities in the polymers networks and also the absence of unreacted monomers; the weak endothermic effect was observed to be attributable to molecular relaxation which is affected the glass transition process by causing changes in the Tg and Cp values during successive heating runs [105,106]. The influence of the varied chain length of PEGMEM on the Tg value was observed, however, it is not unambiguously linear. The increase in the glass transition temperature with the increase in the co-monomer chain length occurred only in the order of polymers P3–P5. The similar trend was observed in the studies of HD and TG.

### 4.10. PXRD

The diffraction patterns of the co-polymers P1–P5 were compared with the pure monomer and initiator to obtain information on the degree of crystallinity of the polymerization products in relation to the initial material. The diffractograms of the P1–P5 co-polymers, presented in Figure 13A, did not exhibit any characteristic distinctive peaks due to the periodicity of the crystalline phases. Instead, the X-ray scattering profiles included two broad diffraction peaks, indicating poorly crystalline or a non-crystalline material. The broad diffractions lines may appear in nano-sized materials, characterized by a very low crystallinity [41,107]. According to the estimations on the basis of the Scherrer equation, the average crystallite size between 1.85 and 1.92 nm may indicate polymers with nanocrystallites that are embedded at large distances away from one another in an amorphous matrix [38]. Therefore, the tested polymer products P1–P5 revealed the features of an amorphous system. The crystallite size which was obtained by XRPD is much smaller than the corresponding values that were reported in the DLS analysis. The size of the crystals which were measured by XRPD are indicative, and the aggregates may be composed of crystalline domains and therefore, the crystallite size is sometimes smaller than the aggregate size is [108].

The maxima in the diffractograms of the P1–P5 co-polymers occurred at approximately the same angles, which suggests that increasing of the length of the polyoxyethylene glycol chain did not result in a shift of the peaks, however, the use of PEGs as co-monomers in the synthesis of the P1–P5 copolymers resulted a small shift of diffractions peaks, approximately of 0.5 degrees, both for the peak 1 and 2, as compared to literature [109,110]. Changes in the intensity of the diffractograms of the tested samples may indicate differences in the molecular structure [111]. Specifically, this may suggest that co-polymers P2 and P3 have similar molecular structures.

## 5. Conclusions

In this study, the thermosensitive co-polymers P1–P5 of NIPA and co-monomers PEGMEM with different chains lengths (Mn 200–1500) were successfully synthesized via surfactant-free precipitation polymerization in an aqueous environment at 70 °C. The applied conductometric measurements may be used as a tool, facilitating the estimation of the subsequent stages of the polymerization process, and can be further evaluated for kinetic studies. The influence of the length of the PEGMEM chain on the physicochemical properties of the obtained co-polymers was investigated. The studies of hydrodynamic diameter confirmed that synthesized co-polymers tend to aggregate and are in a nanoscale. The increase in the number of polyoxyethylene groups in the chain results in the increased size of the polymer particle. The estimated LCST values in the HD measurements vs. temperature were in the range of the physiological temperature of 31–33 °C. No linear relationship between the PEGMEM chain length of the co-polymers P1–P5 and the LCST value was observed. The particle size distribution by intensity at 18 °C and 45 °C indicates a polymodal population at lower and monomodal at higher temperatures, which confirms the presence of molecular aggregation. The PDI values occurring in the range of 0.42–0.65 and 0.13–0.26 in the temperatures below and above the LCST, respectively, pointed out a decreasing polydispersity with increasing temperature. The recorded ZP results confirm the formation of particles with a negative surface charge throughout the entire temperature range of 18–45 °C with a tendency to increase the colloidal stability at temperatures that are above the LCST.

According to both the ATR-FTIR and ^1^H NMR results in the polymerization reaction, the copolymers of NIPA and PEGMEM have been synthesized. The analysis of the ^1^H NMR spectra at two different temperatures below and above the LCST show changes in the structure of the tested copolymers, resulting from various interactions between the proton which originated from the water molecule and the hydrophilic groups of the copolymers. The TG results demonstrate that the all of the co-polymers were thermally stable up approximately 240 °C, independent of the chain length. The DSC data confirm the effect of the PEGMEM chain length on the TG results. The PXRD measurements suggest that the polymers P1–P5 may possess semicrystalline structures. The assessed systems may be in the future recognized as suitable for controlled drug delivery, according to our former studies which employed similar structures, however, here we are focusing on the properties of the macromolecules and the reaction course as evaluated by the conductometric method.

## Figures and Tables

**Table 1:** Substrates compositions of P1, P2, P3, P4, and P5 nano co-polymers.

Components		Type of Co-Polymer Nanoparticle System				
		P1	P2	P3	P4	P5
Monomer (g)	NIPA	5.0054	5.0987	5.0051	5.0090	5.0180
Anionic initiator (g)	APS	0.5009	0.5082	0.5042	0.5076	0.5011
Co-monomers (g)	PEGMEM (Mn~200)	0.5098	-	-	-	-
	PEGMEM (Mn~300)	-	0.5070	-	-	-
	PEGMEM (Mn~500)	-	-	0.5176	-	-
	PEGMEM (Mn~950)	-	-		0.5057	-
	PEGMEM (Mn~1500)	-	-			0.5112

**Table 2:** The results of the TG and DTG curve analyses of P1–P5.

Type of Polymer Nanoparticle System	T_1_ (°C)	Rate of Mass Loss 1 (% min^−1^)	T_2_ (°C)	Rate of Mass Loss 2 (% min^−1^)	T_3_ (°C)	Rate of Mass Loss 3 (% min^−1^)	*T*_Onset_ (°C)	*T*_Endset_ (°C)	Res. at 760 °C (%)	*T*_1.0wt%_ (°C)
P1	52.3	0.59	274.0	0.50	397.9	9.45	343.0	410.5	6.88	31.8
P2	51.0	0.68	274.9	0.46	396.2	9.68	356.3	408.6	4.38	33.1
P3	48.8	0.64	293.3	0.45	395.7	9.54	361.5	410.3	7.10	31.3
P4	52.0	0.67	289.7	0.50	395.9	9.38	357.3	395.9	7.15	31.7
P5	53.2	0.65	288.1	0.42	397.6	9.99	372.3	411.9	7.10	31.8

**Table 3:** The parameters calculated from XRD data of P1–P5 co-polymer samples: peak position, intensities, and FWHM of selected diffraction peaks for the 2θ = 5°−70°.

Sample	Peak 1 2θ (°)	Int.1 (Arbitrary Units)	FWHM 1	Peak 2 2θ (°)	Int. 2 (Arbitrary Units)	FWHM 2
P1	7.98	20,677	3.39	19.66	16,260	6.12
P2	7.94	19,347	3.32	20.12	14,358	6.03
P3	7.89	19,751	3.19	19.78	14,351	6.01
P4	8.07	18,197	3.44	19.72	16,389	5.63
P5	7.94	21,071	3.37	19.47	15,129	5.81

## Data Availability

The data presented in this study are available on request from the corresponding author.

## Sample Availability

Samples of the compounds P1–P5 are available from the authors.
